# Endocranial volume is variable and heritable, but not related to fitness, in a free-ranging primate

**DOI:** 10.1038/s41598-021-81265-w

**Published:** 2021-02-19

**Authors:** Abigail E. Colby, Clare M. Kimock, James P. Higham

**Affiliations:** Department of Anthropology, New York University, New York, NY 10003 USA

**Keywords:** Biological anthropology, Heritable quantitative trait, Behavioural ecology

## Abstract

Large relative brain size is a defining characteristic of the order Primates. Arguably, this can be attributed to selection for behavioral aptitudes linked to a larger brain size. In order for selection of a trait to occur, the trait must vary, that variation must be heritable, and enhance fitness. In this study, we use a quantitative genetic approach to investigate the production and maintenance of variation in endocranial volume in a population of free-ranging rhesus macaques. We measured the endocranial volume and body mass proxies of 542 rhesus macaques from Cayo Santiago. We investigated variation in endocranial volume within and between sexes. Using a genetic pedigree, we estimated heritability of absolute and relative endocranial volume, and selection gradients of both traits as well as estimated body mass in the sample. Within this population, both absolute and relative endocranial volume display variation and sexual dimorphism. Both absolute and relative endocranial volume are highly heritable, but we found no evidence of selection on absolute or relative endocranial volume. These findings suggest that endocranial volume is not undergoing selection, or that we did not detect it because selection is neither linear nor quadratic, or that we lacked sufficient sample sizes to detect it.

## Introduction

Variation in relative brain size across mammals^1–3^ is thought to be attributable to selection in response to different social and/or ecological conditions favoring adaptations for specific behavioral attributes that are linked to a larger brain^1,2,4–7^. In mammals, larger relative brain size is linked to extended longevity and enhanced cognitive abilities, such as increased information-processing capacity, enhanced memory, innovation, and learning^8–10^. A large relative brain size is a defining characteristic of primates^11^, who display a variety of complex social and ecological behaviors. In anthropoid primates, diverse kinds of sophisticated cognitive abilities are widespread, and relative brain size is positively correlated with total lifespan^12,13^. However, a large brain is energetically expensive and linked to reduced fecundity and extended ontogenetic periods^14,15^. As such, primates likely occupied ecological niches that favored enhanced cognition and facilitated the evolution of slower life history.

There are numerous hypotheses to explain primate encephalization (increased brain size). The two that have received the most attention are the social brain hypothesis and the ecological brain hypothesis. The social brain hypothesis argues that large relative brain size in primates is an adaptation for living in large social groups. It reasons that group-living requires greater information-processing capacity to enhance an organism’s ability to maintain a large social network and acquire mates, although which mating system is the most cognitively demanding is debated^6,16–18^. The ecological brain hypothesis posits that large brain sizes in primates are driven by dietary complexity. In contrast to foliage, fruit and animal matter are patchy in distribution and can be enclosed in complex matrices, such that frugivorous and faunivorous primates rely upon mental maps and tool use to access preferred food^19–22^.

A single driving factor for encephalization is advocated in many hypotheses, however, some researchers have proposed that there is likely to be no single explanation, and that a more integrative approach which combines the various explanations for the evolution of large brain size into a unitary explanatory framework is needed to understand the evolution of brain enlargement in primates^23^. In primates, group living likely evolved in response to food distribution and predation risk^24–26^. Given the close relationship between ecology and sociality, it is possible that encephalization is due to a variety of factors that may differ between taxa as a result of unique ecological conditions. Most research on encephalization and cognition in mammals examines interspecific variation, which offers valuable insights on brain origins^3^. However, most research does not address intraspecific variation or heritability, which are both requirements for natural selection, or if/why living nonhuman primate populations are currently undergoing selection for a larger brain. In this study, we investigate variation and heritability of endocranial volume (as a proxy for brain size) and its relationship to fitness and longevity in a free-ranging population of rhesus macaques (*Macaca mulatta*) to improve our understanding of the evolution of brain size in a nonhuman primate species.

For a trait to be under selection it must be heritable, and brain size has been shown to be heritable in many laboratory and captive animals. Brain size has been shown to be heritable in laboratory mice^27^, laboratory rats^28^, captive hamadryas baboons (*Papio hamadryas*)^29^, captive chimpanzees (*Pan troglodytes*)^30,31^, captive vervet monkeys (*Chlorocebus aethiops sabaeus*)^32^, three-spined sticklebacks (*Gasterosteus aculeatus*)^33^, and guppies (*Poecilia reticulata*)^34^. Most research investigating the heritability of brain size in mammals has been limited to captive and laboratory populations, not wild or free-ranging populations, however, endocranial volume has been shown to be heritable in free-ranging rhesus macaques^35^ and wild red deer (*Cervus elaphus*)^3^. Logan et al.^3^ reported not only that endocranial volume is highly heritable but that it is linked to longevity and lifetime reproductive success in the wild red deer population of the Isle of Rum. Cheverud et al.^35^ estimated heritability of endocranial volume in the same free-ranging rhesus macaque population of Cayo Santiago that we sample in the present study, and reported that it is highly heritable. However, that study used mother–offspring regression and symmetric-differences-squared (SDS) methods to estimate heritability—methods that do not account for additive genetic variance inherited from the paternal lineage, or that account for other fixed and random effects that may influence phenotype^36^. In this study, we estimate heritability in the same population of free-ranging rhesus macaques using a larger sample size and statistical methods that account for additive genetic variance inherited from the paternal lineage.

If a trait is to be under selection it must also present an evolutionary advantage to the survival and reproduction of an individual. However, little is known regarding whether intraspecific variation of brain size is linked to life history and/or fitness in free-ranging or wild taxa. One study that investigated intraspecific variation of brain size in pumpkinseed sunfish (*Lepomis gibbosus*)^37^, found that populations of fish living in more complex ecosystems showed larger relative brain volumes than populations living in simpler environments, suggesting that environmental conditions can select for larger brain sizes in the wild. As noted previously, Logan et al.^3^ reported that larger endocranial volume is linked to longer lifespans and increased lifetime reproductive success in female red deer. These studies suggest that larger brains may be linked to increased longevity and fitness by providing an enhanced ability to navigate a complex environment, leading to selection for larger brain size in mammals. To date, we are unaware of reported research that has investigated intraspecific variation of brain size and links to life history and fitness in a population of nonhuman primates. One reason for the lack of research on heritability and intraspecific variation of brain size in nonhuman primates may be that pedigreed populations with large endocranial volumes are extremely uncommon. The Cayo Santiago population of rhesus macaques offers a unique opportunity to investigate these questions. Heritability and selection in numerous morphological and behavioral traits have been studied in this population^35,38–41^.

The objectives of this study are: *(i)* to investigate inter-individual *variation and sex differences *in absolute and relative endocranial volume; *(ii)* to estimate *heritability* of absolute and relative endocranial volume; and *(iii)* to investigate what types of selection, if any, absolute and relative endocranial volume are under, via *selection gradients*. To address these questions, we measured the endocranial volume of 542 rhesus macaque skeletal specimens of the free-ranging population of Cayo Santiago. This sample included males and females from ten generations. To address inter-individual variation and sex differences, we compared the measured endocranial volumes of male and female specimens. To investigate heritability, we used the available pedigree and a Bayesian animal model^42^ to estimate additive genetic variance in the sample population. To investigate potential associations between variation in the traits and variation in fitness, we estimated selection gradients using two different proxies for fitness: lifetime reproductive success, and longevity.

## Results

### Variation and sex differences

**Figure 1:**
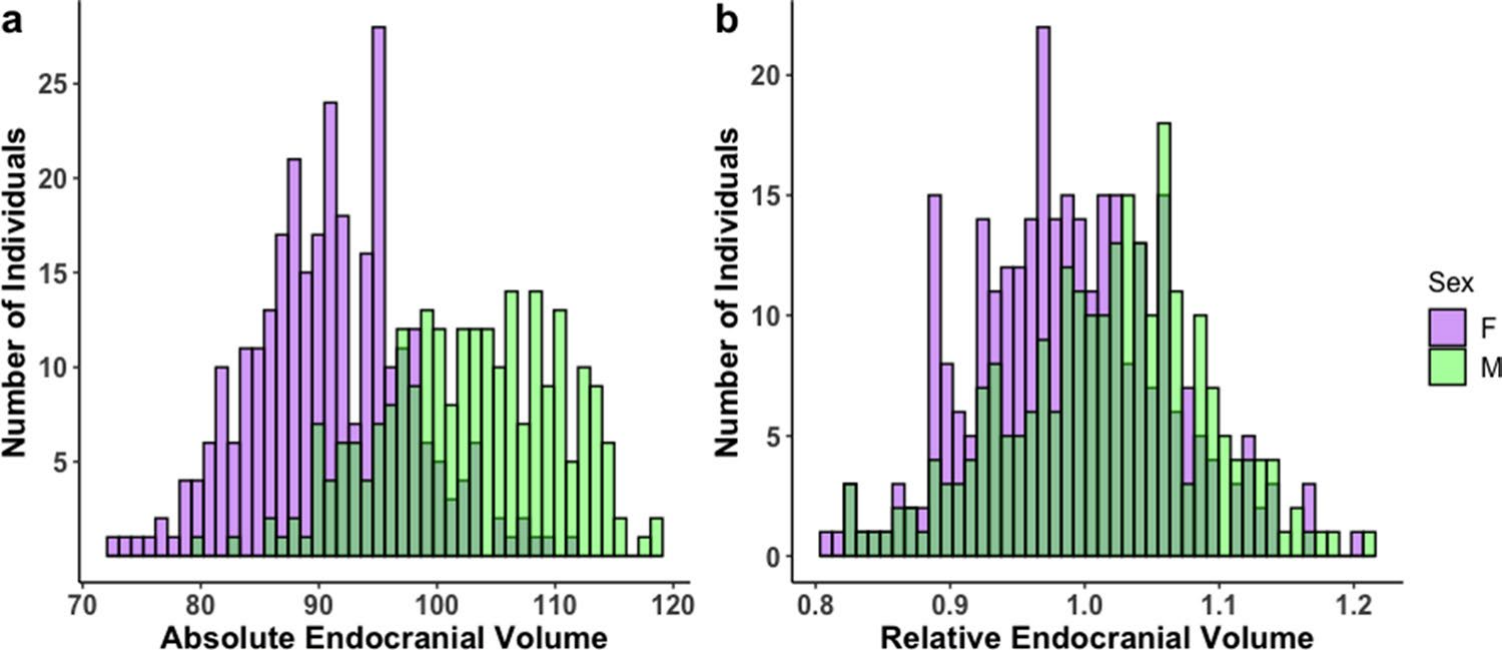
Histogram illustrating variation in absolute endocranial volume (**a**), and relative endocranial volume (**b**) in all specimens included in the study (females = 300, males = 242, total = 542). Relative endocranial volume was calculated as the encephalization quotient (endocranial volume divided by the fitted values of linear model of geometric mean against endocranial volume). Females are depicted in purple and males in green.

We found a substantial amount of variation in absolute endocranial volume within the combined sample of males and females (mean = 96.13 ± 9.02 ml, coefficient of variance (CV) = 9.39), among females (mean = 90.93 ± 6.53 ml, CV = 7.18), and among males (mean = 102.57 ± 7.38 ml, CV = 7.20). The mean values of female and male absolute endocranial volume were significantly different (p =  < 0.001), which can be partially explained by moderate body size dimorphism in rhesus macaques, and the correlation between brain size and body size across mammals (Table 1, Fig. 1a). There was a substantial amount of variation in relative endocranial volume within the pooled sample of males and females (mean = 1.00 ± 0.07, CV = 7.30), among females (mean = 0.99 ± 0.07, CV = 7.13), and among males (mean = 1.01 ± 0.07, CV = 7.26). The mean values of female and male relative endocranial volume were significantly different (p < 0.001) (Table 1, Fig. 1b).

**Table 1:** Variation and sex differences in absolute endocranial volume and relative endocranial volume (n = 300 females, n = 242 males).

	Mean ± SD (ml)	Coefficient of variance (CV)	p-value
**Absolute endocranial volume**			**< 0.001**
All	96.13 ± 9.02	9.39	
Females	90.93 ± 6.53	7.18	
Males	102.57 ± 7.38	7.20	
**Relative endocranial volume**			**< 0.001**
All	1.00 ± 0.07	7.33	
Females	0.99 ± 0.07	7.15	
Males	1.01 ± 0.07	7.24	

Relative endocranial volume is reported as the encephalization quotient (endocranial volume measurements divided by the fitted values of a linear model of geometric mean against endocranial volume). A bolded p-value depicts statistical significance (p < 0.05) calculated by t-tests comparing sexes.

### Heritability

Absolute endocranial volume was highly heritable (*h*^2^ = 0.660; 95% Bayesian credible interval (CI) = 0.444–0.816). Only a small amount of variation was estimated as being related to maternal effects (< 0.001; CI < 0.001–0.078). Relative endocranial volume was also highly heritable (0.631; CI = 0.408–0.820). Maternal effects accounted for a small portion of trait variation (< 0.001; CI < 0.001–0.062). This suggests that individual variation in endocranial volume is highly influenced by additive genetic variance (Table 2a).

**Table 2:** (**2a**) Heritability results for absolute endocranial volume and relative endocranial volume (n = 300 females, n = 242 males). Heritability and random effects with a highest distribution posterior interval (HDPI) of 0.3 or greater and fixed effects with a pMCMC value of < 0.05 are bolded. (**2b**) Posterior means of each variance component for endocranial volume with 95% credible intervals (CI) in parentheses.

2a	Absolute endocranial volume (n = 542)	Relative endocranial volume (n = 542)
Heritability (HDPI)	**0.688 (0.445–0.824)**	**0.634 (0.401–0.818)**
**Random effects**		
Maternal ID (HDPI)	< 0.001 (< 0.001–0.079)	< 0.001 (< 0.001–0.062)
Residual (HDPI)	**0.230 (0.150–0.512)**	**0.361 (0.173–0.576)**
**Fixed effects**		
Geometric mean (pMCMC)		**< 0.001**
Sex (pMCMC)	**< 0.001**	**< 0.001**
DIC	955.0546	961.1919

2b	Absolute endocranial volume (n = 542)	Relative endocranial volume (n = 542)
**Variance components**		
Additive genetic variance (*V*_a_)	0.390 (0.251–0.529)	0.352 (0.201–0.488)
Maternal variance (*V*_m_)	0.0137 (< 0.001–0.045)	0.010 (< 0.001–0.036)
Residual variance (*V*_r_)	0.198 (0.093–0.305)	0.209 (0.098–0.319)

### Selection gradients

**Figure 2:**
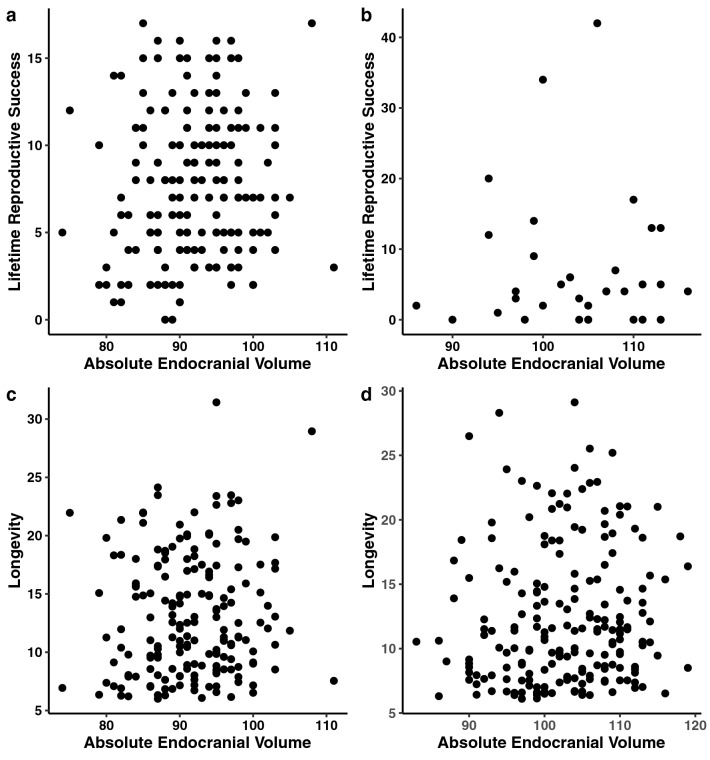
Absolute endocranial volume against: (**a**) lifetime reproductive success (females) (n = 177); (**b**), lifetime reproductive success (males) (n = 34); (**c**) longevity (females) (n = 186); (**d**) longevity (males) (n = 198). Lifetime reproductive success is the number of offspring to survive to one year. Longevity is measured as age at death.

**Figure 3:**
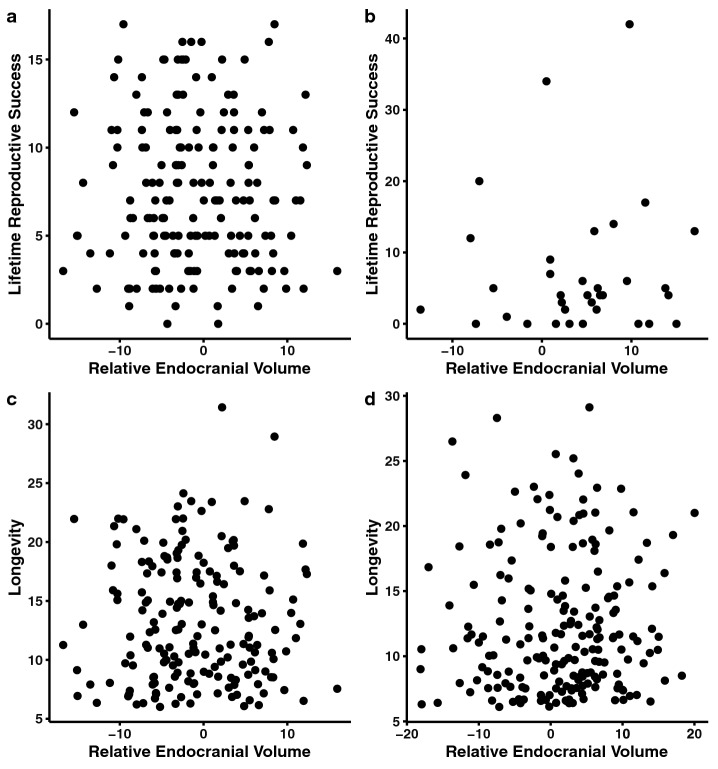
Relative endocranial volume against: (**a**) lifetime reproductive success (females) (n = 177); (**b**), lifetime reproductive success (males) (n = 34); (**c**) longevity (females) (n = 186); (**d**), longevity (males) (n = 198). The x-axis depicts the residuals of a linear model (i.e., endocranial volume ~ geometric mean). Lifetime reproductive success is the number of offspring to survive to one year. Longevity is measured as age at death.

In both sexes, the linear and quadratic models suggest that absolute endocranial volume is unrelated to either longevity or lifetime reproductive success, and therefore, is not undergoing directional, disruptive, or stabilizing selection (Table 3a,b, Fig. 2a–d). Likewise, linear and quadratic models on relative endocranial volume indicate no relationship between trait values and fitness, and therefore, is not currently undergoing directional, disruptive, or stabilizing selection (Table 4a,b, Fig. 3a–d). Alternatively, body size does seem subject to selection. Both linear and quadratic models on relative endocranial volume did detect significant relationships between geometric mean and lifetime reproductive success in females but not males, suggesting that females may be undergoing directional selection for a larger body size, or stabilizing selection for an intermediate body size (Table 4a,b).

**Table 3:** Linear (3a) and quadratic (3b) selection gradients (GLMs) for absolute endocranial volume in females and males.

3a	Females	Males
Absolute endocranial volume		
**Lifetime reproductive success**	n = 177	n = 34
Selection gradient	*β* = 0.007 ± 0.007	*β* = − 0.001 ± 0.033
	*t* = 1.093	*t* = − 0.038
	*p* = 0.276	*p* = 0.970
**Longevity**	n = 186	n = 198
Selection gradient	*β* = 0.003 ± 0.005	*β* = 0.004 ± 0.004
	*t* = 0.675	*t* = 1.048
	*p* = 0.501	*p* = 0.296

3b	Females	Males
Absolute endocranial volume		
**Lifetime reproductive success**	n = 177	n = 34
Selection gradient (quadratic term)	γ_*ii*_ = − 0.0003 ± 0.004	γ_*ii*_ = − 0.003 ± 0.004
	*t* = − 0.417	*t* = -0.753
	*p* = 0.678	*p* = 0.457
Linear term	*t* = 0.469	*t* = 0.751
	*p* = 0.640	*p* = 0.459
**Longevity**	n = 186	n = 198
Selection gradient (quadratic term)	γ_*ii*_ = < 0.001 ± < 0.001	γ_*ii*_ = − 0.0002 ± 0.0005
	*t* = 0.062	*t* = − 0.400
	*p* = 0.950	*p* = 0.690
Linear term	*t* = − 0.030	*t* = 0.445
	*p* = 0.976	*p* = 0.657

Selection gradients are the estimate ± the standard error. *β* indicates the linear selection gradient, γ_*ii*_ the quadratic selection gradient, t the t-value, and p the p-value.

**Table 4:** Linear (4a) and quadratic (4b) selection gradients (GLMs) for relative endocranial volume in females and males.

4a	Females	Males
Relative endocranial volume		
**Lifetime reproductive success**	n = 177	n = 34
Selection gradient	*β* = 0.003 ± 0.007	*β* = 0.005 ± 0.035
	*t* = 0.559	*t* = 0.149
	*p* = 0.577	*p* = 0.883
Geometric mean	*t* = 2.425	*t* = − 0.644
	**p = 0.016**	*p* = 0.542
**Longevity**	n = 186	n = 198
Selection gradient	*β* = 0.001 ± 0.005	*β* = 0.003 ± 0.004
	*t* = 0.304	*t* = 0.835
	*p* = 0.762	*p* = 0.405
Geometric mean	*t* = 1.563	*t* = 1.341
	*p* = 0.120	*p* = 0.181

4b	Females	Males
Relative endocranial volume		
**Lifetime reproductive success**	n = 177	n = 34
Selection gradient (quadratic term)	γ_*ii*_ = − 0.0003 ± 0.0007	γ_*ii*_ = − 0.003 ± 0.004
	*t* = − 0.395	*t* = − 0.674
	*p* = 0.6935	*p* = 0.505
Linear term	*t* = 0.422	*t* = 0.680
	*p* = 0.6736	*p* = 0.502
Geometric mean	*t* = 2.414	*t* = − 0.555
	**p = 0.0168**	*p* = 0.583
**Longevity**	n = 186	n = 198
Selection gradient (quadratic term)	γ_*ii*_ = < 0.001 ± < 0.001	γ_*ii*_ = − 0.0002 ± 0.0004
	*t* = 0.077	*t* = − 0.563
	*p* = 0.939	*p* = 0.574
Linear term	*t* = 0.041	*t* = 0.599
	*p* = 0.951	*p* = 0.550
Geometric mean	*t* = 1.560	*t* = 1.396
	*p* = 0.121	*p* = 0.164

Selection gradients are the estimate ± the standard error. Statistical significance (p < 0.05) is indicated in bold. *β* indicates the linear selection gradient, γ_*ii*_ the quadratic selection gradient, t the t-value, and p the p-value.

## Discussion

In this study, we used a quantitative genetic approach to investigate the presence, production, and maintenance of variation in endocranial volume in the Cayo Santiago macaques. We found that absolute and relative endocranial volume are variable, within and between sexes, and are highly heritable. However, we found no evidence that endocranial volume is a predictor of fitness and, therefore, no evidence of selection on endocranial volume in females or males in this population.

We found evidence of sexual dimorphism in brain size in which males have significantly larger absolute and relative endocranial volumes than females. This finding is consistent with a previous study on the same population^43^, as well as studies on chimpanzees^31,44^, ground squirrels^45^, pocket gophers^46^, three-spined sticklebacks^47^, wild red deer^3^, and humans^48^. In general, this brain size dimorphism is associated with behavioral dimorphisms observed between males and females. For example, greater aptitudes in spatial reasoning in males that may serve as an advantage for mate acquisition and, therefore, a mating strategy that may be maintained via intrasexual selection^43,45,46^. However, selection gradient analyses indicated no evidence of selection on endocranial volume in males.

We found that a large amount of the phenotypic variation in absolute endocranial volume and relative endocranial volume can be explained by additive genetic variance, with very little contribution from maternal ID, and with the remaining variance attributable to unidentified environmental factors. Our results are consistent with those of other studies investigating brain size or endocranial volume in other taxa, which include captive baboons^39^, captive vervet monkeys^32^, rhesus macaques^35^, and wild red deer^3^ in that all studies report high heritability of the trait.

We did not find links between endocranial volume and lifetime reproductive success or longevity, and therefore did not find evidence of selection on endocranial volume. However, we did find relationships between lifetime reproductive success and body size, in both linear (directional selection for larger body size) and quadratic models (stabilizing selection), indicating selection for larger body size and/or intermediate body size in female rhesus macaques. Large body size is associated with high dominance rank in free-ranging vervet monkeys^49^, free-ranging Japanese macaques (*Macaca fuscata*)^50^, and captive rhesus macaques^51^. High rank is also linked to reduced interbirth interval in rhesus macaques^52^ and offspring survival in bonnet macaques (*Macaca radiata*)^53^. We were unable to control for rank in our statistical analyses because rank data was not available for a sufficient number of individuals, and so our results may reflect the influence of rank, not body size, on fitness, if rank and body size were correlated in the dataset. We did not find evidence of selection on absolute or relative endocranial volume in males. This finding is consistent with that of Logan et al.^3^, who found no link between endocranial volume and lifetime breeding success, annual fecundity, or longevity in male red deer. However, our sample size of males for measures of lifetime reproductive success was small, and results may differ if a larger sample size was available.

We propose three interpretations of the results that show a lack of evidence for selection. First, this population has not been under selection for brain size at this time. There is considerable variation in the trait, but this could all be within the fitness peak for the trait, such that the associated genotypic variation is not under selection. Second, selection is present in this population, but our selection gradient analyses are not powerful enough to detect selection with our current sample sizes. Lastly, selection is occurring in this population, however, it is neither linear nor quadratic. For example, a form of balancing selection that is not detectable using linear or quadratic models might be present and acting on endocranial volume.

In order to understand the evolution of the brain, additional research investigating intraspecific variation, heritability, and, most importantly, evidence of selection in living populations of free-ranging and wild mammals is needed. Future studies should not only address the existence of sexual dimorphism in brain size but assess potential explanations. Additionally, most research investigating brain size heritability is limited to captive and laboratory populations. An effort to investigate more wild and free-ranging populations is necessary in order to understand how additive genetic variance, as well as other random effects, influence phenotypic variation in brain size. To address selection on brain size, more research investigating the relationship between brain size and proxies of evolutionary fitness in more mammalian taxa, as well as the development and incorporation of statistical methods that can detect forms of selection that are not linear or quadratic, is needed. Additionally, a study in this population that incorporates a larger sample size of specimens for lifetime reproductive success, especially males, is needed. At present, such a sample of males is unavailable due to the life history of rhesus macaques and the relatively recent addition of genetic parentage data relative to the skeletons that have been curated into the Caribbean Primate Research Center skeletal collection.

In conclusion, endocranial volume is variable and heritable but is not related linearly or quadratically to proxies of evolutionary fitness in this free-ranging rhesus macaque population. There is nonetheless evidence for variation and high levels of heritability of this trait in this population. One possibility is that brain size has reached its fitness optimum, and that the observed genetic variation is within the range of the fitness peak. An alternative is that we may have been unable to detect selection due to limitations in statistical approaches and sample sizes. More research investigating intraspecific variation of brain size is important for understanding the origins and evolution of large brain sizes in primates, including humans.

## Materials and methods

### Study site

All permissions to measure crania were given to JH by the Caribbean Primate Research Center. The skeletal samples used in this study are from the Cayo Santiago population of rhesus macaques. Cayo Santiago is a 15.2-hectare island located 1 km off of the southeast coast of Puerto Rico in the Caribbean Sea. In 1938, the colony was established with a population of 409 individuals transplanted from India^54^. The colony is currently maintained by the Caribbean Primate Research Center and it has been monitored continuously since 1956. The Caribbean Primate Research Center maintains databases on behavioral and demographic information as well as genetic parentage information on individuals born after 1985 and skeletal remains have been systematically collected since the 1970s^54–56^. The macaques live in naturally formed social groups. They are, however, provisioned and provided with commercial monkey chow and water ad libitum. Despite the small size of the founding population, the Cayo Santiago macaques are not inbred and show similar genetic diversity to other populations of rhesus macaques^54,57^. The Caribbean Primate Research Center provided pedigree and demographic (maternal ID, matriline, origin, location of death, date of birth, date of death, age at death) data for all individuals used in the study.

The sample (N = 542) included adult females (n = 300) and adult males (n = 242) with intact neurocrania; all specimens were skeletally mature and had reached their adult endocranial volume. Skeletal maturity, as determined by epiphyseal union, occurs at 5.25 years in females and 6.08 years in males^58–60^. Adult endocranial volume is reached at 3.57 years in females and 6.08 years in males^61^. We used all females that were at least 5.25 years old, and all males that were at least 6.08 years old. All individuals were born on Cayo Santiago, however, some individuals died on Cayo Santiago (n = 383) while others were moved during their lifetime and died at the Sabana Seca Field Station (n = 159).

### Endocranial measurements

One of us (AC) measured the endocranial volumes and two postcranial proxies of body size. Endocranial volume was obtained by pouring 2 mm glass beads into the foramen magnum of each specimen and subsequently pouring the beads into a graduated cylinder and recording the estimated volume (ml). Logan et al.^62^ report this method as the most accurate method for estimating endocranial volume. Body size proxies were femoral length and femoral medio-lateral breadth^63^ of each specimen, which are postcranial traits that correlate with body mass in Cercopithecoidea^64^. We calculated a geometric mean based on these measures that served as a proxy for body size in statistical analyses. To assess intra-rater reliability of skeletal measurements, an intraclass correlation analysis was performed (ICC > 0.95).

### Pedigree

A pedigree database, maintained by the Caribbean Primate Research Center, contains information on behavioral dams (identified via observation and available for all individuals) and genetic dams and genetic sires (identified via microsatellite panel and available for all individuals born after 1985)^56^. We pruned the pedigree to include the 542 individuals for which endocranial volume was measured and their relevant relatives (connections between the individuals of known endocranial volume) (R package: MasterBayes)^65,66^. The pruned pedigree included 900 individuals (maternities = 847, paternities = 197, full siblings = 3, maternal siblings = 922, paternal siblings = 172, maternal grandmothers = 746, paternal grandmothers = 197, maternal grandfathers = 60, paternal grandfathers = 47), across ten generations (R package: pedantics)^67^.

In selection analyses, fitness was measured as lifetime reproductive success (number of offspring to survive to one year) and longevity (age at death)^3^. All measures of fitness were extracted from the pedigree and demographic databases.

### Statistical analyses

#### Variation and sex differences

We visualized inter-individual variation by calculating the mean, standard deviation, and coefficient of variance (CV) of absolute and relative endocranial volume. We calculated relative endocranial volume as the encephalization quotient (endocranial volume divided by the fitted values of a linear model in which endocranial volume is the response variable and geometric mean is the predictor variable) to account for the allometric relationship between brain size and body size. We conducted t-tests to examine sex differences in absolute endocranial volume and relative endocranial volume of all specimens in the study (n = 300 females, n = 242 males).

#### Heritability

We used an animal model to estimate narrow-sense heritability (*h*^2^) of absolute and relative endocranial volume of the sample used in this study (R package: MCMCglmm)^66,68,69^. An animal model is a univariate generalized linear mixed model (GLMM) which combines individual phenotypic records and pedigree information to assess the influence of additive genetic and environmental factors toward variation in a phenotypic trait^42,68,70^. All models included female (n = 300) and male (n = 242) specimens derived from Cayo Santiago and the Sabana Seca Field Station. We ran heritability models on scaled values for traits of interest.

We used a stepwise model reduction procedure comparing Akaike’s Information Criterion (AIC) to determine which fixed effects (sex, geometric mean, age at death) and random effects (maternal ID, matriline, birth year, location of death) would be used in the model (R package: lmerTest)^3,71^. Following stepwise model reduction, our final model for absolute endocranial volume included sex as a fixed effect and maternal ID as a random effect. Our final model for relative endocranial volume included sex and geometric mean as fixed effects and maternal ID as a random effect.

We ran the absolute and relative endocranial volume models for 2,550,000 iterations with a burn-in period of 50,000 iterations and a thinning interval of 1000. We used a parameter expanded prior (v = 1, nu = 1, alpha.mu = 0, alpha.v = 1000) in the animal models to investigate absolute endocranial volume and relative endocranial volume^36^. To ensure model convergence, we verified that autocorrelation between samples were less than 0.1 and we visually assessed density plots of the Markov chain Monte Carlo (MCMC) chain^36,66^. We calculated heritability by dividing additive genetic variance by total phenotypic variance (*h*^2^ = *V*_*A*_/*V*_*P*_).

#### Selection gradients

We used selection gradient models to assess whether absolute endocranial and relative endocranial volume predict variation in lifetime reproductive success and longevity^72,73^. We separated female and male data for selection analyses. We ran linear models to detect directional selection and quadratic models to investigate stabilizing and disruptive selection. In absolute endocranial volume models, endocranial volume was included as a predictor variable and fitness measures as response variables. In relative endocranial volume models, endocranial volume was included as a predictor variable and geometric mean as a fixed effect (or covariate), and lifetime reproductive success and longevity as response variables. In linear models, we used untransformed endocranial volume, while in quadratic models we used squared untransformed endocranial volume^74^. In all models, the response variables were mean-standardized (the value of an individual divided by the mean of the sample)^75^. A Gaussian error distribution was used in all models of fitness for both females and males.

In selection analyses, only specimens that were born on Cayo Santiago and died on Cayo Santiago were included. All specimens were included in longevity models (n = 185 females, n = 198 males). However, given that consistent monitoring of the Cayo Santiago colony began in 1956 and assignments of genetic parentage began in 1985, all females in lifetime reproductive success models were born in or after 1956 (n = 177) and all males in lifetime reproductive success models were born in or after 1985 (n = 34)^55,56^. Models on lifetime reproductive success in males failed to meet assumptions (residuals were not normally distributed) unless we square-root transformed lifetime reproductive success values. We present results of models on untransformed data here because response variables should not be transformed in selection gradients^72^. Results of models on transformed data can be found in the Supplemental Materials (Supplemental Tables 1 and 2), but they did not differ qualitatively from the results presented in the main manuscript.

### Ethical statement

This research complied with Animal Behavior Society guidelines for ethical research conduct. No work with live animals took place, and so no ethical approvals were necessary.

## Supplementary Information

Supplementary Information.

## Data Availability

The datasets generated and/or analyzed during the current study, alongside the code for running the analyses, can be found at https://doi.org/10.6084/m9.figshare.13312799.

## References

[1] Healy SD, Rowe C (2007). A critique of comparative studies of brain size. Proc. R. Soc. B Biol. Sci..

[2] Roth G, Dicke U (2005). Evolution of the brain and intelligence. Trends Cogn. Sci..

[3] Logan CJ, Kruuk LEB, Stanley R, Thompson AM, Clutton-Brock TH (2016). Endocranial volume is heritable and is associated with longevity and fitness in a wild mammal. R. Soc. Open Sci..

[4] Dunbar RIM (1992). Neocortex size as a constraint on group size in primates. J. Hum. Evol..

[5] Innocenti GM, Kaas JH (1995). The cortex. Trends Neurosci..

[6] Kaas JH (1995). The evolution of isocortex. Brain. Behav. Evol..

[7] Barton RA, Harvey PH (2000). Mosaic evolution of brain structure in mammals. Nature.

[8] Reader SM, Laland KN (2002). Social intelligence, innovation, and enhanced brain size in primates. Proc. Natl. Acad. Sci..

[9] Sol D, Székely T, Liker A, Lefebvre L (2007). Big-brained birds survive better in nature. Proc. R. Soc. B Biol. Sci..

[10] Benson-Amram S, Dantzer B, Stricker G, Swanson EM, Holekamp KE (2016). Brain size predicts problem-solving ability in mammalian carnivores. Proc. Natl. Acad. Sci. USA.

[11] Cartmill M (2005). New views on primate origins. Evol. Anthropol. Issues News Rev..

[12] Allman J, McLaughlin T, Hakeem A (1993). Brain weight and life-span in primate species. Proc. Natl. Acad. Sci..

[13] González-Lagos C, Sol D, Reader SM (2010). Large-brained mammals live longer. J. Evol. Biol..

[14] Harvey PH, Bennett PM (1983). Evolutionary biology: Brain size, energetics, ecology and life history patterns. Nature.

[15] Aiello LC, Wheeler P (1995). The expensive-tissue hypothesis: The brain and the digestive system in human and primate evolution. Curr. Anthropol..

[16] Kudo H, Dunbar RIM (2001). Neocortex size and social network size in primates. Anim. Behav..

[17] Schillaci MA (2006). Sexual selection and the evolution of brain size in primates. PLoS ONE.

[18] Shultz S, Dunbar RIM (2007). The evolution of the social brain: anthropoid primates contrast with other vertebrates. Proc. R. Soc. B Biol. Sci..

[19] King BJ (1986). Extractive foraging and the evolution of primate intelligence. Hum. Evol..

[20] Barton RA (1996). Neocortex size and behavioural ecology in primates. Proc. R. Soc. Lond. B.

[21] DeCasien AR, Williams SA, Higham JP (2017). Primate brain size is predicted by diet but not sociality. Nat. Ecol. Evol..

[22] Powell LE, Isler K, Barton RA (2017). Re-evaluating the link between brain size and behavioural ecology in primates. Proc. R. Soc. B Biol. Sci..

[23] Dunbar RIM, Shultz S (2017). Why are there so many explanations for primate brain evolution?. Philos. Trans. R. Soc. B Biol. Sci..

[24] Van Schaik CP (1983). Why are diurnal primates living in groups?. Behaviour.

[25] Van Schaik CP, Van Hooff JARAM (1983). On the ultimate causes of primate social systems. Behaviour.

[26] Wrangham RW (1980). An ecological model of female-bonded primate groups. Behaviour.

[27] Atchley WR, Riska B, Kohn LAP, Plummer AA, Rutledge JJ (1984). A quantitative genetic analysis of brain and body size associations, their origin and ontogeny: Data from mice. Evolution.

[28] Riska B, Atchley WR (1985). Genetics of growth predict patterns of brain-size evolution. Science.

[29] Rogers J (2007). Heritability of brain volume, surface area and shape: An MRI study in an extended pedigree of baboons. Hum. Brain Mapp..

[30] Gómez-Robles A, Hopkins WD, Schapiro SJ, Sherwood CC (2015). Relaxed genetic control of cortical organization in human brains compared with chimpanzees. Proc. Natl. Acad. Sci..

[31] DeCasien AR, Sherwood CC, Schapiro SJ, Higham JP (2020). Greater variability in chimpanzee (*Pan troglodytes*) brain structure among males. Proc. R. Soc. B.

[32] Fears SC (2009). Identifying heritable brain phenotypes in an extended pedigree of vervet monkeys. J. Neurosci..

[33] Noreikiene K (2015). Quantitative genetic analysis of brain size variation in sticklebacks: Support for the mosaic model of brain evolution. Proc. R. Soc. B Biol. Sci..

[34] Kotrschal A (2013). Artificial selection on relative brain size in the guppy reveals costs and benefits of evolving a larger brain. Curr. Biol..

[35] Cheverud JM (1990). Heritability of brain size and surface features in rhesus macaques (*Macaca mulatta*). J. Hered..

[36] de Villemereuil, P. Tutorial estimation of a biological trait heritability using the animal model How to use the MCMCglmm R package. (2012).

[37] Axelrod CJ, Laberge F, Robinson BW (2018). Intraspecific brain size variation between coexisting sunfish ecotypes. Proc. R. Soc. B Biol. Sci..

[38] Blomquist GE (2009). Fitness-related patterns of genetic variation in rhesus macaques. Genetica.

[39] Brent LJN (2014). Personality traits in rhesus macaques (*Macaca mulatta*) are heritable but do not predict reproductive output. Int. J. Primatol..

[40] Dubuc C (2014). Sexually selected skin colour is heritable and related to fecundity in a non-human primate. Proc. R. Soc. B Biol. Sci..

[41] Kimock CM, Dubuc C, Brent LJN, Higham JP (2019). Male morphological traits are heritable but do not predict reproductive success in a sexually-dimorphic primate. Sci. Rep..

[42] Kruuk LEB (2004). Estimating genetic parameters in natural populations using the ‘animal model’. Philos. Trans. R. Soc. B.

[43] Falk D, Froese N, Sade DS, Dudek BC (1999). Sex differences in brain/body relationships of Rhesus monkeys and humans. J. Hum. Evol..

[44] Herndon JG, Tigges J, Anderson DC, Klumpp SA, McClure HM (1999). Brain weight throughout the life span of the chimpanzee. J. Comp. Neurol..

[45] Iwaniuk AN (2001). Interspecific variation in sexual dimorphism in brain size in Nearctic ground squirrels (*Spermophilus* spp.). Can. J. Zool..

[46] Towe AL, Mann MD (1995). Habitat-related variations in brain and body size of pocket gophers. J. Hirnforsch..

[47] Kotrschal A, Räsänen K, Kristjánsson BK, Senn M, Kolm N (2012). Extreme sexual brain size dimorphism in sticklebacks: A consequence of the cognitive challenges of sex and parenting?. PLoS ONE.

[48] Ritchie SJ (2018). Sex differences in the adult human brain: Evidence from 5216 uk biobank participants. Cereb. Cortex.

[49] Whitten PL (1983). Diet and dominance among female vervet monkeys (*Cercopithecus aethiops*). Am. J. Primatol..

[50] Mori A (1979). Analysis of population changes by measurement of body weight in the Koshima troop of Japanese monkeys. Primates.

[51] Small MF (1981). Body fat, rank, and nutritional status in a captive group of Rhesus Macaques. Int. J. Primatol..

[52] Sade DS (1976). Population dynamics in relation to social structure on Cayo Santiago. Ybk. Phys. Anthr..

[53] Silk JB, Clark-Wheatley CB, Rodman PS, Samuels A (1981). Differential reproductive success and facultative adjustment of sex ratios among captive female bonnet macaques (*Macaca radiata*). Anim. Behav..

[54] Rawlins RG, Kessler MJ (1986). The Cayo Santiago macaques: History, behavior, and biology.

[55] Kessler MJ, Rawlins RG (2016). A 75-year pictorial history of the Cayo Santiago rhesus monkey colony. Am. J. Primatol..

[56] Widdig A (2016). Genetic studies on the Cayo Santiago rhesus macaques: A review of 40 years of research. Am. J. Primatol..

[57] Widdig A (2017). Low incidence of inbreeding in a long-lived primate population isolated for 75 years. Behav. Ecol. Sociobiol..

[58] Cheverud JM (1981). Epiphyseal union and dental eruption in *Macaca mulatta*. Am. J. Phys. Anthropol..

[59] Turnquist JE, Kessler MJ (1989). Free-ranging Cayo Santiago rhesus monkeys (*Macaca mulatta*): I. Body size, proportion, and allometry. Am. J. Primatol..

[60] Havill LM (2004). Osteon remodeling dynamics in macaca mulatta: Normal variation with regard to age, sex, and skeletal maturity. Calcif. Tissue Int..

[61] Konigsberg L (1990). External brain morphology in rhesus macaques (*Macaca mulatta*). J. Hum. Evol..

[62] Logan CJ, Clutton-Brock TH (2013). Validating methods for estimating endocranial volume in individual red deer (*Cervus elaphus*). Behav. Process..

[63] Jolly, C. *The classification and natural history of Theropithecus (Simopithecus) (Andrew, 1916) baboons of the African Plio-Pleistocene*. (Bull. Brit. Mus. Nat. Hist., 1972).

[64] Delson, E. *et al. Body mass in Cercopithecidae (Primates, mammalia): Estimation and scaling in extinct and extant taxa*. (American Museum of Natural History, 2000).

[65] Hadfield JD, Richardson DS, Burke T (2006). Towards unbiased parentage assignment: Combining genetic, behavioural and spatial data in a Bayesian framework. Mol. Ecol..

[66] Hadfield, J. D. *MCMCglmm Course Notes*. (2016).

[67] Morrissey MB, Wilson AJ (2009). pedantics: An r package for pedigree-based genetic simulation and pedigree manipulation, characterization and viewing: Computer program article. Mol. Ecol. Resour..

[68] Hadfield JD (2010). MCMC methods for multi-response generalized linear mixed models: The MCMCglmm R package. J. Stat. Softw..

[69] Hadfield JD, Nakagawa S (2010). General quantitative genetic methods for comparative biology: Phylogenies, taxonomies and multi-trait models for continuous and categorical characters. J. Evol. Biol..

[70] Wilson AJ (2010). An ecologist’s guide to the animal model. J. Anim. Ecol..

[71] Kuznetsova A, Brockhoff PB, Christensen RHB (2017). lmerTest package: Tests in linear mixed effects models. J. Stat. Softw..

[72] Lande R, Arnold SJ (1983). The measurement of selection on correlated characters. Evolution.

[73] Morrissey MB, Sakrejda K (2013). Unification of regression-based methods for the analysis of natural selection. Evolution.

[74] Stinchcombe J, Agrawal A, Hohenlohe P, Arnold S, Blows M (2008). Estimating nonlinear selection gradients using quadratic regression coefficients: Double or nothing?. Evolution.

[75] Matsumura S, Arlinghaus R, Dieckmann U (2012). Standardizing selection strengths to study selection in the wild: A critical comparison and suggestions for the future. Bioscience.

