# Supplementary material for: Endocranial volume is variable and heritable, but not related to fitness, in a free-ranging primate
Source: Sci Rep. 2021 Feb 19;11:4235. doi: 10.1038/s41598-021-81265-w (PMC7895985; doi:10.1038/s41598-021-81265-w)
Supplement: Supplementary file 1 — Supplementary Information. [file 41598_2021_81265_MOESM1_ESM.docx]

**Endocranial volume is variable and heritable, but not related to fitness, in a free-ranging primate**

Abigail E. Colby^1^, Clare M. Kimock^1^, & James P. Higham^1^

*^1^Department of Anthropology, New York University, New York, NY 10003*

| **1a** | **linear** |
| --- | --- |
| **absolute endocranial volume** |  |
| *lifetime reproductive success* | n = 34 |
| selection gradient | *β* = <0.001 ± 0.015  *t* = 0.025  *p* = 0.980 |
| *longevity* | n = 198 |
| selection gradient | *β* = 0.002 ± 0.002  *t* = 1.0232  *p* = 0.219 |
| **1b** | **quadratic** |
| **absolute endocranial volume** |  |
| *lifetime reproductive success* | n = 34 |
| selection gradient (quadratic term) | γ*_ii_* = -0.001 ± 0.002  *t* = -0.603  *p* = 0.551 |
| selection gradient (linear term) | *t* = 6.03  *p* = 0.551 |
| *longevity* | n = 198 |
| selection gradient (quadratic term) | γ*_ii_* = <-0.001 ± <0.001  *t* = -0.273  *p* = 0.785 |
| selection gradient (linear term) | *t* = 0.325  *p* = 0.745 |

**Supplemental Table 1.** **1a.** Linear selection gradients (GLMs) for absolute endocranial volume in males. All response variables were square-root transformed in these models. Selection gradients are the estimate ± the standard error. Statistical significance (p<0.05) is indicated in bold. *β* indicates the linear selection gradient, t the t-value, and p the p-value. **1b.** Quadratic selection gradients (GLMs) for absolute endocranial volume in males. Selection gradients are the estimate ± the standard error. Statistical significance (p<0.05) is indicated in bold. γ*_ii_* indicates the quadratic selection gradient, t the t-value, and p the p-value.

| **2a** | **linear model** |
| --- | --- |
| **relative endocranial volume** |  |
| *lifetime reproductive success* | n = 34 |
| selection gradient | *β* = 0.002 ± 0.016  *t* = 0.151  *p* = 0.881 |
| geometric mean | *t* = -0.445  *p* = 0.659 |
| *longevity* | n = 198 |
| selection gradient | *β* = 0.002 ± 0.002  *t* = 1.011  *p* = 0.313 |
| geometric mean | *t* = 1.388  *p* = 0.167 |
| **2b** | **quadratic model** |
| **relative endocranial volume** |  |
| *lifetime reproductive success* | n = 34 |
| selection gradient (quadratic term) | γ*_ii_* = -0.001 ± 0.002  *t* = -0.546  *p* = 0.589 |
| selection gradient (linear term) | *t* = 0.552  *p* = 0.585 |
| geometric mean | *t* = -0.373  *p* = 0.712 |
| *longevity* | n = 198 |
| selection gradient (quadratic term) | γ*_ii_* = <-0.001 ± <0.001  *t* = -0.440  *p* = 0.660 |
| selection gradient (linear term) | *t* = 0.483  *p* = 0.630 |
| geometric mean | *t* = 1.428  *p* = 0.155 |

**Supplemental Table 2.** **2a.** Linear selection gradients (GLMs) for relative endocranial volume in males. All response variables were square-root transformed in these models. Selection gradients are the estimate ± the standard error. Statistical significance (p<0.05) is indicated in bold. *β* indicates the linear selection gradient, t the t-value, and p the p-value. **2b.** Quadratic selection gradients (GLMs) for relative endocranial volume in males. Selection gradients are the estimate ± the standard error. Statistical significance (p<0.05) is indicated in bold. γ*_ii_* indicates the quadratic selection gradient, t the t-value, and p the p-value.
